# Low- and No-Calorie Sweetener (LNCS) Presence and Consumption among the Portuguese Adult Population

**DOI:** 10.3390/nu13114186

**Published:** 2021-11-22

**Authors:** María González-Rodríguez, Marina Redruello-Requejo, María de Lourdes Samaniego-Vaesken, Ana Montero-Bravo, Ana M. Puga, Teresa Partearroyo, Gregorio Varela-Moreiras

**Affiliations:** 1 Departamento de Ciencias Farmacéuticas y de la Salud, Facultad de Farmacia, Universidad San Pablo-CEU, CEU Universities, Urbanización Montepríncipe, Alcorcón, 28925 Madrid, Spain; m.gonzalez202@usp.ceu.es (M.G.-R.); m.redruello@usp.ceu.es (M.R.-R.); l.samaniego@ceu.es (M.d.L.S.-V.); amontero.fcex@ceu.es (A.M.-B.); anamaria.pugagimenezazca@ceu.es (A.M.P.); t.partearroyo@ceu.es (T.P.); 2 Grupo USP-CEU de Excelencia “Nutrición para la Vida (Nutrition for Life)”, ref: E02/0720, Alcorcón, 28925 Madrid, Spain

**Keywords:** low- and no-calorie sweeteners, artificial sweeteners, additives, food groups, processed foods, Portuguese population

## Abstract

The use of low and no-calorie sweeteners (LNCS) in food and beverages has become increasingly common in the development and reformulation of products to reduce energy derived from added sugars. Our aim was to identify the presence and consumption of LNCS through food and beverages according to consumption patterns in a representative sample (*n* = 256) of the Portuguese adult population. The study had a descriptive cross-sectional observational design and was based on the application of a Food Frequency Questionnaire. Overall, it was found that 4.1% of the foods and 16.7% of the beverages consumed by the Portuguese adult population contained LNCS. Food groups mostly contributing to LNCS consumption were non-alcoholic beverages such as soft drinks and juices (34.2%); milk and dairy products (16.5%); appetizers such as chips (8.6%); sugars and sweets such as chocolates, candies, or chewing gums (6.1%); meat and derivative products (2.2%); cereals and derivatives (1.2%) and canned fruits (1.2%). Main LNCS consumed were acesulfame-K, sucralose, and aspartame, single or combined, although their prevalence of use differs greatly among foods, beverages, or tabletop sweeteners. In conclusion, LNCS were found across a wide variety of products available in the Portuguese market and their prevalence of inclusion in the diet of the population evidences the need to develop more studies on the evolution of LNCS intake and its impact on the full dietary model and health. Consequently, these food additives should be included in food composition databases and, periodically, updated to reflect the recurrent reformulation strategies adopted by the food industry in its efforts to reduce the energy contribution of added sugars.

## 1. Introduction

World sugar consumption has tripled in the last 50 years, and this increase is expected to continue, mainly in the so-called emerging countries [1]. Currently, sugars are one of the most controversial components in our diet, since a high intake is considered a risk factor for the development of obesity, one of the greatest epidemics of the 21st century [2] along with other non-communicable diseases like diabetes, obesity, cardiovascular disease, tooth decay, etc. [3]. For this reason, most public health authorities worldwide recognize that there is an excessive sugar consumption by the population and, as a result, different policies have been implemented to encourage its reduction [3]. One of the most relevant tools implemented by the food industry has been the use of low- and no-calorie sweeteners (LNCS) as sugar substitutes through formulation or reformulation of foods. Therefore, although in the last decade there have been numerous scientific investigations evaluating their safety, nutritional aspects as well as risk-benefit ratio analysis of LNCS as food ingredients/additives are being conducted, there is still controversy and misinformation about them [4]. LNCS are food additives widely used as sugar substitutes to sweeten foods and beverages all over the world, since they mimic the taste of sugar, presenting the advantage that they are used in quantities that do not increase the caloric content of the food [5,6].

In 2015, the World Health Organization (WHO) [7] published a recommendation to reduce free (or added) sugar intake to less than 10% of the total energy intake (TEI), both in adults and children. In addition, they stated that a reduction of free sugars below 5% of the TEI could have additional health benefits. Specifically, the WHO defines as “free sugars” those monosaccharides and disaccharides added to food by the manufacturer, the cook, or the consumer, plus the sugars naturally present in honey, syrups, and fruit juices. Therefore, according to this definition, “free sugars” could be considered as “added sugars” [7]. In a recent study conducted in Portuguese consumers (*n* = 1010) [8] most subjects were unaware of these WHO recommendations and had difficulties in identifying and categorizing nutritive or LNCS sweetener-related ingredient names, regardless of using information about sugar frequently and considering that information was very important to stay healthy, thus emphasizing the strong need for further health-related policies aimed to decrease sugar intakes. Indeed, data from the “National Food, Nutrition and Physical Activity Survey of the Portuguese Population (IAN-AF 2015–2016)” revealed that globally this population complies with the general WHO recommendation of less than 10%TEI, but would exceed the conditional ones, with mean daily intake of added sugars at 32.1 g/d and of free sugars at 35.3 g/d, contributing 6.8 and 7.5 % of TEI, respectively. However, mean daily intakes and percentage of energy from either added or free sugars peaked close to or even exceeded the WHO general recommendations in children aged 5–9 years (9.6 TEI% from added and 10.6 TEI% from free sugars) and adolescents aged 10–17 years (9.5 TEI% from added and 10.5 TEI% from free sugars) [9]. These findings have also been described in other neighboring countries such as Spain [10], indicating a possible upward trend in the consumption of sugars mainly by the younger generations. However, several studies show that children prefer sweeter foods than adults and that individual preferences wane over time [11,12], with more recent generations of adults demonstrating no evidence of stronger preferences for sweet than earlier generations of adults [13] despite today’s higher sugar intakes [1].

Mechanisms underlying the age-related decline in sweet preferences remain unknown, while several studies show that sensitivity to sweetness and liking or intake of sweet-tasting products are weakly correlated [14]. There is evidence that it is energetic density that drives food preference and not sweet taste itself [15], as brain mechanisms for food reward and appetite evolved under pressures to protect us from scarcity [16]. This may represent a potential confounding factor when assessing the impact of added sugars on consumption of sweetened products and may also result in the use of LNCS failing to perpetuate a significant decrease in added sugar intake. In fact, dissociating energy from sweet taste may alter expectations for sweetness in foods and, potentially, food-seeking patterns and diet quality [15]. Furthermore, there are several control mechanisms known to modulate the desire for sweet-tasting products (alliesthesia, sensory-specific satiety, post-ingestive hormonal changes, etc.) [15] which can also be potentially modified when substituting sugars for LNCS. Either way, the current obesogenic environment offering ultra-palatable foods, many of which are high in added sugars, is also precipitating inappropriate intakes leading to the obesity pandemic we suffer nowadays.

Formulation and reformulation are some of the mechanisms that the food industry can implement in order to meet the energy and nutrient intake objectives of the current population by adapting the nutritional composition of food [17]. When doing so, concentration of different nutrients (usually fats, sugars or salt) may be changed depending on the objective. The great problem of reducing the sugar content in foods is that it does not only provide a sweet taste, but it may also modify other properties such as texture, moisture or the capacity to prevent microbial growth during food processing and storage [18]. As there are many functions involved, sugar reformulation is quite challenging. Nonetheless, reformulation may not entail a significant reduction in energy in food and beverages, and it might even have the opposite effect to that desired; foods reformulated with sweeteners tend to be perceived as “healthier”, so there is a tendency to consume them in excess [5]. In addition, there is some scientific evidence in animals [19] that excessive consumption of LNCS may lower the satiety threshold and alter glucose homeostasis mechanisms, with possible links to the development of metabolic syndrome and obesity [20]. However, there is a lack of evidence for this hypothesis in humans.

For all the above, the aim of this research was to identify the presence and consumption of LNCS through food and beverages according to the dietary patterns in a representative sample of the Portuguese adult population.

## 2. Materials and Methods

### 2.1. Study Design and Sample

The study had a cross-sectional descriptive and observational design and was based on the application of a series of personal and individual surveys to each of the participants at street level. The study protocol was approved by the Clinical Research Ethics Committee of CEU San Pablo University, with the approval code 447/20/27. The selection of the individuals to be surveyed was carried out in a random and stratified manner, taking into account the representativeness of the five regions corresponding to the NUTS II areas of Portugal (Center, Lisbon Metropolitan Area, North, Alentejo, and Algarve) [21]. As inclusion criteria were considered: (I) any person over 18 years of age who lives in Portugal with a minimum residence time of 1 year and (II) any person who agreed to sign the informed consent. According to the population residing in Portugal over 18 years of age, assuming a global error of ±6.2% for a normal asymptotic confidence interval with a bilateral 95.5% correction for finite populations, considering an infinitive universe and estimation of equally probable categories (*p* = *q* = 50%), a sample of 256 people were included in the study. Once the study target was selected, a first contact or pilot test was planned in order to guarantee an optimal design and the adequacy of the questionnaires to be applied, before proceeding to their final validation and the launch of the work on the global field.

### 2.2. Data Collection

Fieldwork comprising the recruitment and survey processes was carried out from mid-October to December 2019 by the market research company Madison MK (TELECYL^TM^, Valladolid, Spain); while data processing was carried out in the laboratories of the Nutrition and Food Sciences Area of the Faculty of Pharmacy of the CEU San Pablo University (Madrid, Spain). The surveys were programmed into a computer-aided web interviewing (CAWI) application that allowed its online completion by each participant assisted by an interviewer via telephone. The computerized data collection allowed for the automatic control of the quality of the information collected in the application (allowed response ranges, filters and jumps in the questions, incorrect information notices, etc.). Additionally, continuous controls and supervision were carried out in order to guarantee the correct understanding and completion of the surveys.

The surveys that were carried out on the study population were the following: (I) a questionnaire on socio-demographic data and (II) a Food Frequency Questionnaire (FFQ) adapted from a validated short questionnaire on frequency of dietary intake [22]. The FFQ (Supplementary Material) registered the individual average consumption of 65 food and beverage items over the previous year in order to account for seasonal variation. It was specifically designed by the research team of the CEU San Pablo University to assess the consumption of foods that contain sweeteners in the Portuguese market, and it follows the same methodology that was used for the evaluation of LNCS consumption amongst the Spanish adult population [23]. For each processed product consumed, participants were also asked to specify, in detail, its “type” and “brand” so that the product label could be consulted to analyze and register the absence or presence—and type—of LNCS.

### 2.3. Statistical Analysis

A descriptive analysis of the sample and its main quantitative variables, expressed through centralization and dispersion parameters, was conducted. The Shapiro–Wilk normality test was performed on all samples, and in this way the normality of the distribution was verified. To verify the homogeneity of the medians, the Mann–Whitney test was used to determine the comparisons between the medians and to detect significantly different pairs. To verify if the frequencies observed in each category were compatible with the independence between both variables, Pearson’s chi-square test was used. The level of statistical significance was established at *p* ≤ 0.05. All analyses were completed using the SPSS v.27.0 program (IBM Corp., Armonk, NY, USA).

## 3. Results

The description of the Portuguese population sample is shown in Table 1. Considering the results obtained for the total population (*n* = 256), it can be observed that there was a parity between both genders included: men (*n* = 126) and women (*n* = 130). Regarding age groups, there was a significantly higher proportion of women (65.5%) in the mid-life ages (36–55 years) vs. other age-groups (*p* ≤ 0.001) who participated in the study, while men presented higher percentage for the age group of 18–35 (57.3%) and over 55 years (55.4%) compared with the age group of 36–55 years. Likewise, it can be noted that there was a significantly higher percentage of males (61.9%) with non-compulsory secondary studies than females (38.1%; *p* ≤ 0.001) while women had a higher frequency of university studies (70.9%) than men (29.1%; *p* ≤ 0.001). Analyzing the NUTS II areas, in the metropolitan area of Lisbon there was a higher proportion of women who answered the survey (67.1%) than men (32.9%) (*p* ≤ 0.001).

**Figure 1:**
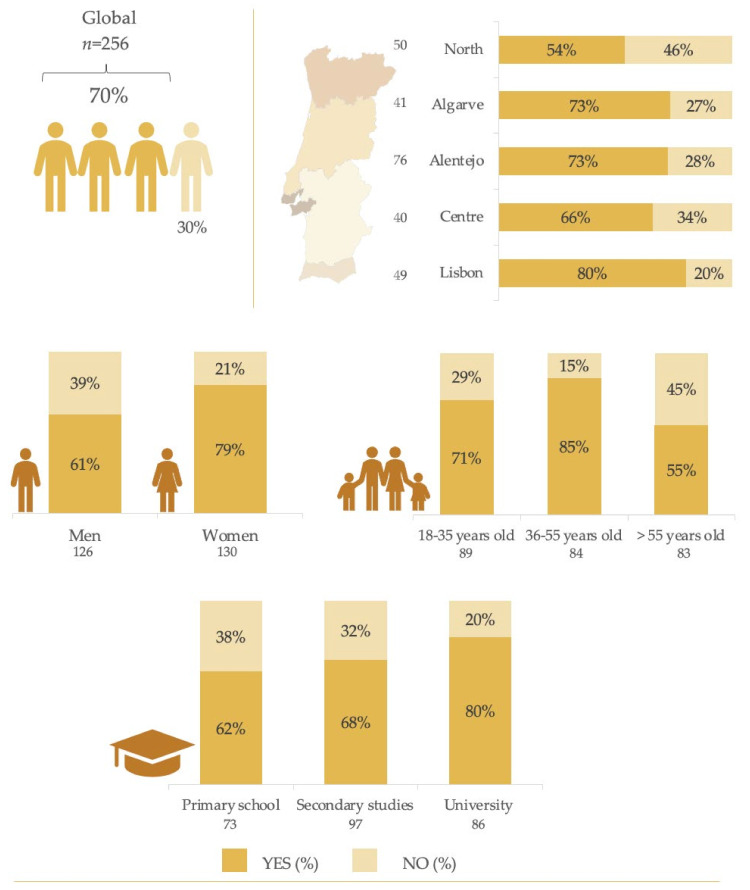
Consumption of low- and no-calorie sweeteners (LNCS) by the Portuguese population.

Figure 1 shows the consumption of LNCS observed in the sample population and detailed for the socio-demographic variables considered. Overall, 70.3% of the total sample consumed food products contained LNCS. Groups for which consumption was found to be significantly higher (*p* ≤ 0.005) were women (79.2%), population aged 36–55 years (84.5%), those with university studies (80.2%), and residents in the Metropolitan Area of Lisbon (80.0%).

Analyzing the products that accounted for the consumption of LNCS, it was found that 4.1% of the foods and 16.7% of the beverages consumed by the Portuguese adult population contained LNCS. Table 2 shows the distribution of the presence of LNCS in each of the food groups. LNCS were consumed in food and beverages included in the groups of non-alcoholic beverages (34.2%), milk and dairy products (16.5%), appetizers (8.6%), sugars and sweets (6.1%), meat and meat products (2.2%), cereals and derivatives (1.2%), and canned fruits (1.2%). No LNCS were identified amongst the other food groups. Interestingly, no significant differences were found when stratifying consumption by gender, nor in their distribution among food groups.

It should be noted that the presence of sweeteners is not homogeneous throughout all foodstuffs that make up each food group. We consider that, in the context of dietary patterns, it is relevant to highlight both the presence and absence of LNCS, and consequently Table S1 lists all the food and beverage products that make up each group and the presence or absence of LNCS for each one.

**Figure 2:**
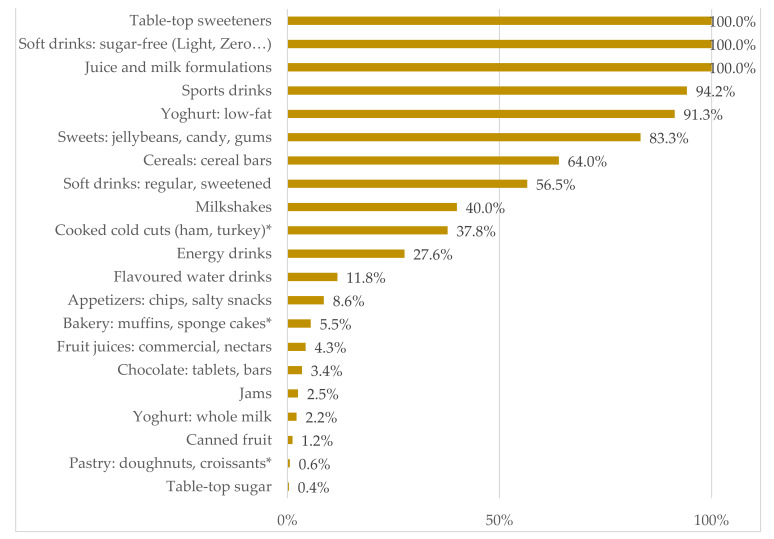
Presence of low- and-no-calorie sweeteners (LNCS) in all food products contributing to LNCS consumption. * LNCS found was sorbitol, for which the declared use was not as a sweetener but as humectant or as stabilizer.

Figure 2 shows in further detail the presence of LNCS in each product consumed. All tabletop sweeteners consisted of LNCS, evidencing that these have displaced caloric sweeteners, which is consistent with the objective of reducing their energy contribution. Similarly, the totality of low or sugar-free soft drinks also included LNCS as sugar substitutes. Following this trend, other non-alcoholic beverages such as juice and milk formulations (100%) and sports drinks (94.2%) should be mentioned. The rest of the non-alcoholic beverages consumed have a lower but still relevant presence of LNCS, mainly soft drinks (56.5%) and milkshakes (40.0%).

As for food products other than beverages, a high presence of LNCS is also observed in low-fat yoghurts (91.3%), sweets, candies and chewing gums (83.3%) or cereal bars (64.0%). The remaining food products that contributed to the consumption of LNCS did so in less than 50%, indicating that at least one out of two lacked LNCS.

Presence and type of LNCS included in each food product is detailed in Table 3. Sucralose (E-955) and acesulfame-K (E-950) were the most frequently used LNCS, found in the composition of 11 and 10 different foods products, respectively. These were then followed by aspartame (E-951) found in seven products, cyclamate (E-952) and steviol glycosides (E-960) in six and sorbitol (E-420) in five. Conversely, food products that included the greatest variety of LNCS in their compositions were sweets, candies, and gums (12 different LNCS), sweetened or regular soft drinks (6 LNCS), tabletop sweeteners (5 LNCS), low-fat yoghurts (5 LNCS) and sugar-free soft drinks (4 LNCS). It should be noted that combinations of several LNCS in a product are frequent but do not necessarily include all the LNCS listed here simultaneously.

**Figure 3:**
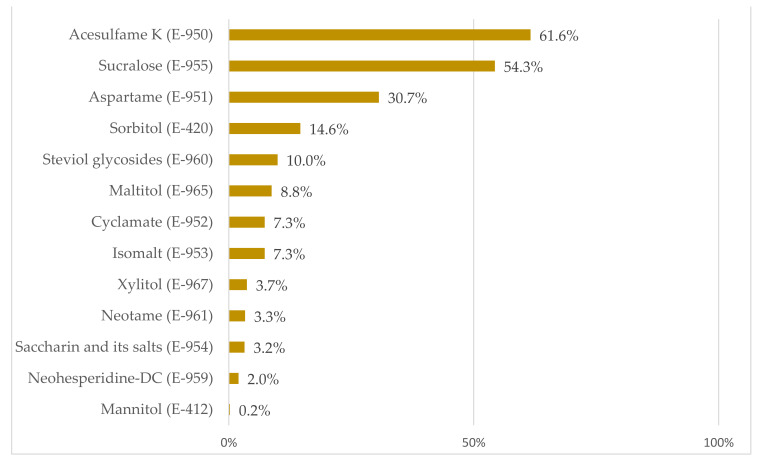
Proportion of low- and no-calorie sweeteners (LNCS) consumed by the Portuguese population.

Figure 3 shows the prevalence of the different types of LNCS found in the evaluated products consumed by the Portuguese population, expressed as a percentage of foodstuffs consumed with that particular LNCS with respect to the total products consumed with LNCS. The most declared LNCS in the different food groups studied were acesulfame K (E-950) and sucralose (E-955) with a proportion of 61.6% and 54.3%, respectively, followed by aspartame (E-951) with 30.7%, and sorbitol (E-420) with 14.6%. The rest of the LNCS showed a presence lower than 10%.

## 4. Discussion

To the best of our knowledge, this is the first study designed to provide a semi-quantitative estimation of LNCS intake in main food and beverage groups consumed by a representative sample of the Portuguese adult population. Unlike sugar consumption, which is almost routinely assessed nowadays, updated information on LNCS intake and its distribution among food groups is almost inexistent in Portugal. Available studies on this matter are restricted to one publication from 2020 [24] assessing the occurrence of three LNCS (acesulfame K, aspartame, and saccharin) by chromatography of different non-alcoholic beverages obtained from the Portuguese market. Based on these data, the exposure of the Portuguese adolescent and adult population was estimated and while it was deemed safe, the authors state that “besides non-alcoholic beverages there are other minor sources of artificial sweeteners in the diet that should also be considered”. As demonstrated in the present work, the intake of LNCS from food products other than beverages should indeed be considered, since some products such as low-fat yogurts, sweets, chocolates, or cereal bars present these additives with a frequency almost similar to that of non-alcoholic beverages and thus cannot be regarded as minor sources of LNCS anymore. Overall, our results are consistent with a recent review by Russell et al. [25] where major products contributing to LNCS intakes worldwide were soft drinks, dairy products (mainly yoghurts), confectionary (including chewing gum), tabletop sweeteners and juices.

Due to the reasons mentioned above, findings reported in the present manuscript will be compared with a recent study of similar methodology developed in a representative sample of the Spanish adult population [23], owing to the similarities between these two countries since their sociocultural and food patterns are part of the so-called Mediterranean diet and associated lifestyle. Results on the overall high consumption levels observed confirm the great level of acceptance of these types of additives among the Portuguese population; although in comparison with the Spanish scenario, a 10% lower prevalence of consumption was observed for the Portuguese population. In any case, consumption trends according to the sociodemographic variables considered are common to both populations, where the greatest consumers were women, those in mid-life ages (36–55 years), with university studies and residents in urban areas. In the review by Russell et al. [25], LNCS consumption trends were also associated with higher education level, socio-economic status and with the female gender, which may be related to modern lifestyles and dietary patterns derived from efforts to control the intake of added sugars and facilitate body weight management. Consistently, Drewnowski et al. [26] also found LNCS use to be more prevalent among the U.S. population with a lower burden of obesity and related chronic disease, particularly women, 45–65 years old, non-Hispanic whites, US-born adults, college graduates and with higher household incomes.

The presence of LNCS in the products consumed by the Portuguese sample population is also slightly lower, with 4.1% of the foods (vs. 4.5% in Spain) and especially in the case of beverages with 16.7% (vs. 22.3% in Spain). Specifically, sources of LNCS intake for the Spanish population were non-alcoholic beverages (36.1%); sugars and sweets (14.2%); milk and dairy products (7.0%); meat and derivatives (5.1%); cereals and derivatives (4.3%); appetizers such as chips (1.7%); and sauces and condiments (1.0%) [23]. After these results, it can be stated that the main source of LNCS for the Portuguese and Spanish population coincide in that they were non-alcoholic beverages, with a presence of LNCS greater than 30% for both countries. However, within this group some differences were found, since in Portugal the presence of LNCS in regular soft drinks doubles (56.5% vs. 24%) while decreasing in flavored water drinks (11.8% vs. 86.0%), in energy drinks (27.6% vs. 41.8%) and in commercial juices or nectars (4.3% vs. 15.9%).

As for food products other than beverages, LNCS presence doubles in milk and milk products consumed in Portugal, with low-fat yoghurts and milkshakes showing a 30% increase in the presence of LNCS compared to those consumed in Spain. This presence increased as well in appetizers (8.6% vs. 1.7%) consumed in Portugal and was also identified in canned fruits (1.2%), pastries such as doughnuts and croissants (0.6%) and, most surprisingly, in tabletop sugar (0.4%); something that had not been observed for the Spanish sample.

When we assessed the types of LNCS declared in products consumed by the Portuguese population, the most frequent were acesulfame K and sucralose with a prevalence of 61.6% and 54.3% respectively, followed by aspartame with a prevalence of 30.7% and sorbitol with a 14.6%; although it should be noted that the declared use of the latter was sometimes as a humectant or stabilizer. Results described for the Spanish adult population [23] are quite similar, although the presence of salt of aspartame-acesulfame (2.9%) and thaumatin (1.6%) was identified in products marketed in Spain but not in Portugal. Since the prevalence of each LNCS is expressed as a percentage of foodstuffs consumed with that particular LNCS with respect to the total products consumed with LNCS, the addition of the prevalence of each LNCS is an indicator of the use of LNCS blending in the same product, and it has been found that in both countries the practice of combining LNCS is also fairly similar.

Nonetheless, it is necessary to highlight the great variability in the predominance of the use of one or another LNCS depending on the food category in which this consumption occurs: beverages, foods other than beverages, or tabletop sweeteners. To this source of variability is now added the one observed in the present study for two markets as *a priori* connected as the Portuguese and Spanish ones, united in the Iberian Peninsula by the Mediterranean diet.

It has already been mentioned that there are practically no further studies assessing the presence of LNCS in food products marketed across Europe. Most of these studies focus on assessing whether consumption is safe—not exceeding the acceptable daily intake levels (ADI)—and thus only evaluate the presence and consumption of the major LNCS. Hence, their results are not entirely comparable. However, an Italian study from 2014 [27] found that non-alcoholic beverages, tabletop sweeteners, and food supplements were the major contributors to LNCS intake. The most consumed sweeteners were acesulfame K, aspartame, and cyclamate; but it should be noted that polyols were not evaluated. Another study conducted in Belgium in 2009–2010 [28], which only assessed the presence of five LNCS (acesulfame K, saccharin, cyclamate, aspartame, and sucralose), revealed that soft drinks (44%) and beers (12%) together accounted for more than half of the total supply of sweetened foods. In this case, aspartame was the most commonly used sweetener (34%) either alone or in combination with acesulfame K, followed by saccharin (24%) and cyclamate (22%); but the presence of other LNCS here identified as highly prevalent, such as sorbitol or steviol glycosides, remained unknown.

The continuous and rapid formulation changes adopted by the food industry make assessments of the presence and intake of LNCS obsolete in a short time, as manufacturers may substitute different amounts and types of sweeteners in a product that maintains its name. An updated database of declared LNCS in foods and beverages marketed in Spain [29] found that in only two years, from 2017 to 2019, food groups with the highest presence of LNCS (soft drinks, fruit juices and nectars, yogurts and fermented milk, and chewing gum and candies) coincided in their distribution, while LNCS presence increased significantly in other minor contributors, such as bakery and pastry products (16% vs. 8%), supplement and meal replacers (9% vs. 4%), breakfast cereals and bars (3% vs. 1%), and ready-to-eat meals (2% vs. 1%). In addition, a higher frequency of addition was found for sorbitol, sucralose, acesulfame K, mannitol, and xylitol [29].

Outside Europe, one country that is consistently working towards the evaluation of LNCS intake is Chile, which recently obliged manufacturers to declare LNCS content in food labeling. As a result, intake assessment is simplified and provides reliable data. Most recent results affirm that LNCS consumption levels were safe [30] and groups with the highest presence of LNCS were non-alcoholic beverages (38.2%), dairy products (28.8%), sweets and other desserts (15.6%), cereal products (14.5%), and processed fruits (2.9%). Sucralose and steviol glycosides were the most widely used LNCS, present, either alone or combined with others, in 73.5% and 39.7% of the LNCS-containing products, respectively, while the use of saccharin and cyclamate was low [31]. However, it should be noted that there are only six authorized LNCS in Chile.

Amongst the main strengths of our work is the assessment of LNCS intake though both beverage and food products, in contrast to several other studies that focus on the former. In addition, a thorough analysis of the nutrition labeling of each product reported by the sample population added reliability and representativeness to the data collection. However, we cannot overlook other limitations inherent in the use of dietary assessments such as underreporting derived from participant and recall biases. Other strengths that have enabled us to provide a comprehensive overview of LNCS consumption across the country are the use of a nationally representative sample that includes five geographical areas (NUTS II areas), as well as different age ranges. This allowed us to focus on food categories and subcategories that are representative of foods most commonly consumed. It is important to highlight that we have not conducted a comprehensive study of the presence of LNCS in the entire Portuguese market, but only in those products consumed by the sample of adults considered. Finally, we are aware that we have conducted an observational study at a specific point in time and that these results represent a snapshot in time which, due to the constant reformulation procedures carried out by the food industry, may change in a matter of months.

## 5. Conclusions

The present research shows that, currently, LNCS can be found in an extensive variety of products available in the Portuguese market. A resulting prevalence of inclusion in the diet of 70% of the Portuguese adult population evidences the need to develop further studies on the evolution of LNCS intake and its impact on the full dietary model and health. The main LNCS used by the Portuguese food industry are acesulfame K (E-950), sucralose (E-955), aspartame (E-951) and sorbitol (E-420). However, a great variability in LNCS use was observed across food categories: foods, beverages, or tabletop sweeteners. Accordingly, there is a great variability of exposure to each LNCS according to the product from which this consumption is derived.

This is the first work carried out in Portugal to identify, examine, and describe the presence of LNCS in the main groups of food and beverages consumed by a representative sample of the Portuguese population. Although conclusions about the total food supply over time is not warranted, this information should be compiled in food composition databases and periodically updated to reflect the recurrent reformulation strategies adopted by the food industry in its efforts to reduce the energy contribution of added sugars.

## Figures and Tables

**Table 1:** Sociodemographic characteristics of the adult Portuguese sample population.

		Total 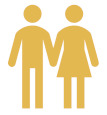		Men 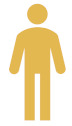		Women 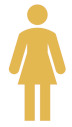	
		*n*	%	*n*	%	*N*	%
**Total Population**		**256**	**100**	**126**	**49.2**	**130**	**50.8**
**Age group** ** 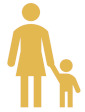 **	**18–35 years**	89	34.8	51	57.3 ^a^	38	42.7 ^a^
	**36–55 years**	84	32.8	29	34.5 ^b^	55	65.5 ^b^
	**>55 years**	83	32.4	46	55.4 ^a^	37	44.6 ^a^
**Education level** ** 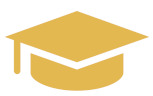 **	**Primary or less**	73	28.5	41	56.2	32	43.8
	**Secondary studies**	97	37.9	60	61.9 ***	37	38.1
	**University**	86	33.6	25	29.1 ***	61	70.9
**Occupational status** ** 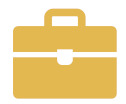 **	**Working**	187	73.0	94	50.3	93	49.7
	**Retired**	33	12.9	16	48.5	17	51.5
	**Other: unemployed,** **students, housewives**	36	14.1	16	44.4	20	55.6
**Geographical** **distribution** **(NUTS II areas)** ** 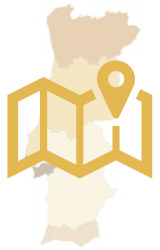 **	**North**	50	19.5	30	60.0	20	40.0
	**Algarve**	49	19.1	28	57.1	21	42.9
	**Alentejo**	40	15.6	18	45.0	22	55.0
	**Centre**	41	16.0	25	61.0	16	39.0
	**Lisbon Metropolitan Area**	76	29.7	25	32.9 ***	51	67.1

Data expressed as median (interquartile range). Different superscript letters indicate statistically significant differences between ages, *p* ≤ 0.001 (Pearson’s chi-square test), *** *p* ≤ 0.001 with respect to women (Pearson’s chi-square test).

**Table 2:** Presence of low and no-calorie sweeteners (LNCS) in food groups consumed by the Portuguese population.

	Presence of LNCS	
Food Group	Yes (%)	No (%)
Beverages: non-alcoholic	34.2	65.8
Milk and dairy products	16.5	83.5
Appetizers	8.6	91.4
Sugar and sweets	6.1	93.9
Meat and derivative products	2.2	97.8
Canned fruit	1.2	98.8
Cereals and derivatives	1.2	98.8
Beverages: alcoholic	0	100
Eggs	0	100
Fish and shellfish	0	100
Fruits	0	100
Nuts and seeds	0	100
Pulses	0	100
Ready-to-eat meals	0	100
Sauces and condiments	0	100
Vegetables	0	100

**Table 3:** Presence and type of low- and-no-calorie sweeteners (LNCS) in all food products contributing to LNCS consumption.

Food Product	%	*N*	LNCS
Tabletop sweeteners	100.0	5	Acesulfame K (E-950) Aspartame (E-951) Saccharin and its salts (E-954) Steviol glycosides (E-960) Sucralose (E-955)
Soft drinks: Low or sugar-free (Light, Diet, Zero…)	100.0	4	Acesulfame K (E-950) Aspartame (E-951) Cyclamate (E-952) Sucralose (E-955)
Juice and milk formulations	100.0	2	Acesulfame K (E-950) Sucralose (E-955)
Sports drinks	94.2	3	Acesulfame K (E-950) Steviol glycosides (E-960) Sucralose (E-955)
Yoghurt: low-fat	91.3	5	Acesulfame K (E-950) Aspartame (E-951) Cyclamate (E-952) Neotame (E-961) Sucralose (E-955)
Sweets: jellybeans, candy, chewing gum	83.3	12	Acesulfame K (E-950) Aspartame (E-951) Cyclamate (E-952) Isomalt (E-953) Lactitol (E-966) Maltitol (E-965) Mannitol (E-421) Polyglycitol syrup (E-964) Saccharin and its salts (E-954) Sorbitol (E-420) Sucralose (E-955) Xylitol (E-967)
Cereals: cereal bars	64.0	3	Maltitol (E-965) Mannitol (E-421) Sorbitol (E-420)
Soft drinks: Regular (sweetened)	56.5	6	Acesulfame K (E-950) Aspartame (E-951) Cyclamate (E-952) Steviol glycosides (E-960) Neohesperidine-DC (E-959) Sucralose (E-955)
Milkshakes	40.0	2	Acesulfame K (E-950) Sucralose (E-955)
Cooked cold cuts (ham, turkey)	37.8	1	Sorbitol (E-420) *
Energy drinks	27.6	1	Acesulfame K (E-950)
Flavoured water drinks	11.8	2	Aspartame (E-951) Sucralose (E-955)
Appetizers: snacks, chips	8.6	1	Aspartame (E-951)
Bakery: muffin, sponge cakes	5.5	1	Sorbitol (E-420) *
Fruit juices: commercial, nectars	4.3	3	Cyclamate (E-952) Steviol glycosides (E-960) Sucralose (E-955)
Chocolate: tablets, bars	3.4	1	Maltitol (E-965)
Jams	2.5	1	Steviol glycosides (E-960)
Yoghurt: whole milk	2.2	2	Acesulfame K (E-950) Sucralose (E-955)
Canned fruits	1.2	2	Cyclamate (E-952) Saccharin and its salts (E-954)
Pastry: doughnuts, croissants	0.6	1	Sorbitol (E-420) *
Tabletop sugar	0.4	1	Steviol glycosides (E-960)

* LNCS found was sorbitol, for which the declared use was not as a sweetener but as humectant or as stabilizer. %: Percentage of food products containing any LNCS; No: number of distinct LNCS found in each food subgroup.

## Data Availability

The data presented in this study are available on request from the corresponding author.

## Supplementary Materials

The following are available online at https://www.mdpi.com/article/10.3390/nu13114186/s1, Table S1. Food groups contributing to Low- and no-calorie sweeteners (LNCS) consumption: detailed list of food products consumed with and without LNCS; Food Frequency Questionnaire (FFQ).
